# Comparison of Different Contrast Agents in Detecting Cardiac Right-to-Left Shunt in Patients with a Patent Foramen Ovale during Contrast-Transthoracic Echocardiography

**DOI:** 10.1155/2017/6086094

**Published:** 2017-12-03

**Authors:** Enfa Zhao, Gesheng Cheng, Yushun Zhang, Yang Li, Yingli Wang

**Affiliations:** ^1^Department of Structural Heart Disease, The First Affiliated Hospital of Xi'an Jiaotong University, Xi'an 710061, China; ^2^Department of Cardiology, Xianyang Hospital of Yan'an University, Xianyang 712000, China

## Abstract

The aim of this study is to evaluate the ability of two different contrast agents to detect cardiac right-to-left shunting in patients with a patent foramen ovale during contrast transthoracic echocardiography and transesophageal echocardiography. Eighty-four patients who had migraines or experienced cryptogenic stroke were prospectively enrolled. Contrast echocardiography of the right portion of the heart was performed using an injection of either (i) 8 ml of agitated saline, 1 ml of blood, and 1 ml of air (ASB) or (ii) 4 ml of vitamin B_6_ and 6 ml of sodium bicarbonate solution (VSBS). All patients underwent contrast echocardiography with different contrast agents successively before undergoing transesophageal echocardiography. The diagnostic sensitivity of VSBS and ASB for cardiac shunting diagnosis was 94.23% and 78.85%, respectively. The diagnostic sensitivity in the VSBS group was significantly higher than that in the ASB group (*χ*^2^ = 5.283, *P* = 0.022). The observed semiquantitative shunt grading suggests that the positive rate in the VSBS group was higher than that in the ASB group (*Z* = −1.998, *P* = 0.046). The use of vitamin B_6_ and sodium bicarbonate solution as a TTE contrast agent yielded a high sensitivity compared with ASB. However, further trials with large sample size are required to confirm this finding.

## 1. Introduction

A patent foramen ovale (PFO) is a remnant of normal fetal circulation that remains open after birth and is present in approximately 25% of adults [1]. PFO has long been considered to be of no clinical significance; however, in recent years, numerous studies have suggested that PFO-right-to-left shunting (RLS) is related to a wide array of disease processes, such as migraine headaches, transient ischemic attack (TIA), cryptogenic stroke, decompression sickness, and platypnea-orthodeoxia syndrome [2–4]. Therefore, it is important to identify PFO-RLS and to determine the degree of shunting severity. PFO-RLS can be detected using three different modalities: contrast-transthoracic echocardiography (c-TTE), contrast-transcranial Doppler (c-TCD), or contrast-transesophageal echocardiography (c-TEE). TEE is currently considered to be the “gold standard” for the diagnosis of PFO. However, technical limitations associated with the procedures are unavoidable, including patients intolerance of the TEE probe or an inability to perform the standard Valsalva maneuver due to the presence of the endoscope in the esophagus under local anesthesia [5]. Additionally, TEE was designed to be semi-invasive, which resulted in discomfort and stress for patients during the examination. These barriers to use have widely reduced the application of TEE in clinical practice. A previous study reported that c-TCD has a high sensitivity and a favorable specificity in detecting PFO-RLS when compared to TEE [6]. Unfortunately, c-TCD is unable to differentiate cardiac from pulmonary right-to-left shunts and provides no data on the shape and size of the defect [7]. However, previous study concluded that contrast TCD has a sensitivity and specificity of 97% and 98%, respectively, compared to contrast TEE as the reference [8]. Furthermore, color flow imaging (CFI) using TTE was shown to be an insensitive (28%) technique for detecting PFO-RLS [9]. Currently, the detection and semiquantitative assessment of PFO-RLS mainly rely on c-TTE. The contrast agents that are currently widely used for the diagnosis of RLS include agitated saline (AS) either alone or with an autologous blood mixture (ASB) [10–13]. Using the acoustic properties of air-filled microbubbles, the contrast agents detect and diagnose PFO-RLS. However, the preparation and injection processes involved in this process are complex and tedious. Vitamin B_6_ and sodium bicarbonate solution as a c-TTE right heart contrast agent has also been used in clinical practice without noticeable side effects [14, 15]. The aim of our study was to determine whether the use of a vitamin B_6_ and sodium bicarbonate solution as a contrast agent results in improved detection of PFO-RLS during c-TTE compared with ASB. Furthermore, we compared the diagnostic sensitivity of VSBS and ASB for cardiac RLS diagnosis with that of TEE, the gold standard.

## 2. Methods

### 2.1. Patient Population

We performed a prospective study that included 84 patients (40 men and 44 women, mean age 41 ± 16  [18–65] years) who were highly suspected of having a PFO. Patients who were referred to our echocardiography laboratory after an episode of TIA, cryptogenic stroke, unexplained cerebral infarction, or migraine headache, between 15 November 2014 and 15 March 2015, were enrolled. The study was approved by the Institutional Clinical Ethics Committee of the First Affiliated Hospital of Xi'an Jiaotong University and was performed in accordance with the CONSORT 2010 guidelines and in accordance with the Declaration of Helsinki (1964). All patients or their relatives provided written informed consent. Previous potential intracranial or extracranial, cardiac and extracardiac malformations and lacunar infarction were ruled out using magnetic resonance imaging or computed tomography. Patients who experienced an acute infection period, serious heart and renal insufficiency, atrium fibrillation, serious heart valve disease, blood hypercoagulable state, superior vena cava and right atrial thrombosis, or cognitive dysfunction and those who were unable to undergo the Valsalva maneuver during c-TTE were also excluded.

### 2.2. TTE Imaging and TEE Examination

**Figure 1:**
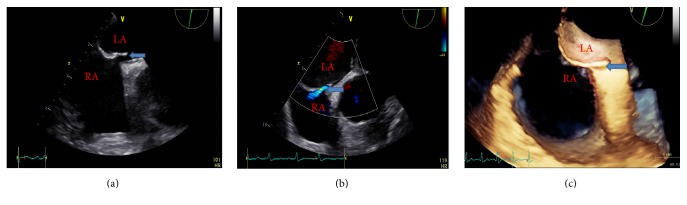
Transesophageal echocardiography demonstrating the interatrial septum. (a) Two-dimensional transesophageal echocardiography showing a “slit-like” communication between the left and right atria, which was diagnosed as a PFO. (b) Color flow mapping of the spontaneous PFO left-to-right shunt. (c) Detection of PFO by real-time three-dimensional transesophageal echocardiography. The tunnel-like configuration between the septum primum and the septum secundum, which indicates the presence of a PFO (blue arrow). LA = left atrium; RA = right atrium.

**Figure 2:**
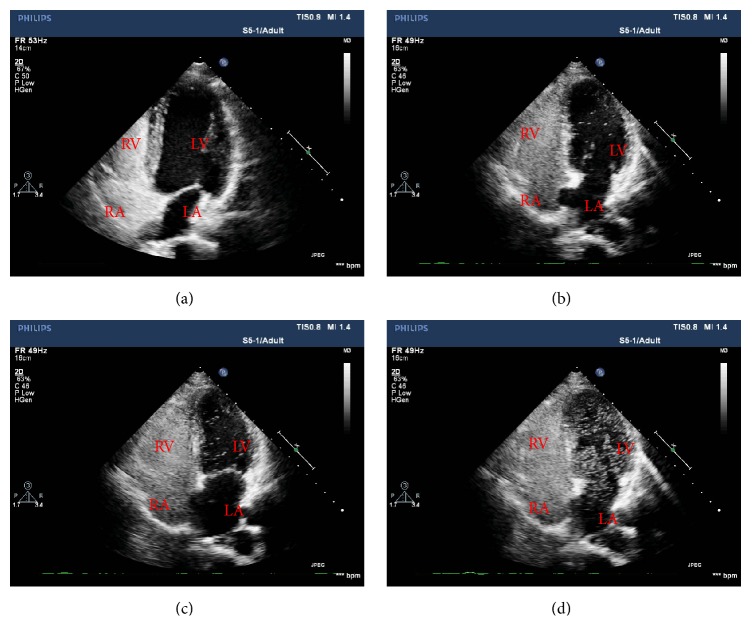
Semiquantitative grading of a patent foramen ovale-right-to-left shunt by transthoracic echocardiography using the bubble test. (a) No right-to-left shunt. (b) Mild right-to-left shunt (1–10 microbubbles in the left atrium). (c) Moderate right-to-left shunt (11–30 microbubbles in the left atrium). (d) Extensive right-to-left shunt (more than 30 microbubbles in the left atrium or left atrial opacity). LA = left atrium; RA = right atrium; LV = left ventricle; RV = right ventricle.

The c-TTE bubble study was conducted by an experienced sonographer using the Philips iE33 imaging systems equipped with a S5-1 (1–5 MHz) probe. TEE was performed using the same system fitted with a 2.9–8 MHz multifrequency probe. Half an hour before the TEE procedure, all patients received 2% lidocaine mucilage for oropharynx anesthesia. The probe was rotated within 45°–110° to clearly display the septum primum and septum secundum as well as to obverse whether an opened PFO and RLS existed both in two-dimensional, three-dimensional, and color Doppler ultrasonography (Figure 1). To ensure maximal diagnostic yield, a standard apical four-chamber view was performed with the administration of contrast agents. Gain settings were adjusted to optimize the visualization of the interatrial septum and valvular structures. The ASB contrast agent, including 8 ml of saline solution, 1 ml of air, and 1 ml of autologous blood, was agitated at least 10 times to enhance the backscatter of the ultrasound beam and to achieve good dilution using two 10 ml syringes that were connected by a 3-way stopcock to exchange the air-saline mix [9, 10, 13]. The VSBS contrast agent consisted of 4 ml of vitamin B_6_ and 6 ml of sodium bicarbonate solution without agitating. The prepared contrast agent was administered intravenously as a bolus via the left antecubital vein. The Valsalva maneuver can enhance the sensitivity of the detection of RLS [9]. All the procedures were conducted with the Valsalva maneuver. The order in which contrast agents were used (ASB or VSBS) was randomly selected for each patient. Furthermore, each contrast material was administered in 30 min intervals. All procedures involved the capture of 10 consecutive beats. The recordings were analyzed retrospectively by two experienced sonographers who were blinded to clinical histories independently. Each bubble study was performed 3 times and the maximum number of microbubbles that was detected was used. The semiquantitative grading of PFO-RLS was classified according to the maximum number of microbubbles that appeared in the left atrium on a still frame based on previous criteria [13, 16] and was defined according to the following criteria: when no, 1–10 bubbles, 11–30 bubbles, and >30 bubbles (or left atrial opacity) were detected, the RLS was considered to be negative, mild, moderate, and extensive, respectively (Figure 2). Cardiac cycle microbubbles appeared in the left atrium after complete opacification of the right atrium was observed. The origin of RLS was determined according to the time by which left atrial microbubbles appeared. That is, for a developing time between 3 and 5 cardiac cycles, RLS was considered to be the result of a PFO, while, for a developing time of more than 5 cardiac cycles, RLS was considered to be the result of pulmonary arteriovenous malformation [17].

### 2.3. Statistical Analysis

Continuous variables are expressed as the mean ± standard deviation, and categorical variables are reported as counts and percentages. A chi-square test was used to compare the total positive rates between groups. The Mann-Whitney-Wilcoxon test was used to compare semiquantitative shunt grading using two contrast agents. Statistical significance was assumed when the *P* value < 0.05. All data were analyzed using SPSS software (version 18.0.1, SPSS Inc.).

## 3. Results

### 3.1. Baseline Characteristics and Positive Detection Rate of Different Contrast Agents

The baseline characteristics of the population included in this study are shown in Table 1. Among the 84 patients who were given different contrast agents, no patient reported a noticeable adverse event during the procedure or during a 12-hour follow-up period. In total, 52 patients were diagnosed with a PFO. Regardless of the degree of shunting severity, the diagnostic sensitivity of VSBS and ASB was 94.23% and 78.85%, respectively. VSBS yielded a higher sensitivity than did ASB (*χ*^2^ = 5.283, *P* = 0.022) (Table 2). However, there was no statistical difference with respect to specificity between the VSBS and ASB groups (84.38% versus 87.50%, *P* > 0.05). ASB exhibited high concordance with TEE for cardiac RLS diagnosis (*κ* = 0.637). VSBS exhibited higher concordance with TEE for cardiac RLS diagnosis (*κ* = 0.796).

### 3.2. Semiquantitative Shunt Grading

According to the semiquantitative grading of PFO-RLS, the degree of PFO-RLS was categorized into four grades. The specific results of the semiquantitative shunt grading are provided in Table 3. The semiquantitative grading of two different contrast agents indicated that the RLS positive rate in the VSBS group was higher than that in the ASB group (*Z* = −1.998, *P* = 0.046).

## 4. Discussion

To our knowledge, this is the first prospective observational study in which vitamin B_6_ and sodium bicarbonate solution was shown to be a better contrast agent than ASB for detecting PFO-RLS. Our preliminary study indicated that using a vitamin B_6_ and sodium bicarbonate solution as a c-TTE contrast agent yielded a higher sensitivity than that with ASB, without producing noticeable adverse events compared to that observed with TEE, which was used as the reference. It has previously been reported that the diagnostic sensitivity of TTE compared to that of TEE (when used as the reference) ranged from 23% (95% confidence interval [CI]: 11%–38%) to 92% (95% CI: 62%–100%) [18–23] which reflects the use of different contrast agents, different microbubble cutoffs for a positive TTE/TEE, and different cardiac cycle cutoffs for a positive TTE/TEE. In our study, we measured a sensitivity of 94.23% and 78.85% for VSBS and ASB, respectively, which was consistent with previous studies. A recent study showed that the sensitivity and specificity of VSBS were 92.31% and 84.88%, respectively, when TEE was used as the reference. Additionally, in our study, we found no difference with respect to specificity between the VSBS and ASB groups (84.38% versus 87.50%, *P* > 0.05).

Determining whether PFO is clinically significant and merits treatment is determined by the presence of PFO-RLS and the degree of shunt severity [15]. Currently, the detection of PFO-RLS mainly relies on c-TTE. However, the type of contrast agent has a great influence on the detection of PFO-RLS and the degree of shunting [24]. The most widely used contrast agents currently include AS or ASB [25]. It has generally been believed that adding blood to AS can stabilize microbubbles and increase their suspension time in the blood [24]. Similarly, agitated saline with blood can also emulsify the microbubbles and prevent them from dissolving in systemic circulation [12], which facilitates the passage of more microbubbles through the PFO. Meanwhile, mixing blood with agitated saline can result in an increase in the viscosity of the contrast material and can further reduce the rate of injection. Whether the presence of air microbubbles in the blood causes adverse effects remains controversial. Indeed, improper operation will result in large microbubbles and blood clots [26]. However, the contrast agents that are commonly used are heterogeneously mixed. Notably, this requires a complex and cumbersome agent injection, which involves two 10 ml syringes connected by a 3-way stopcock to exchange the air-saline mix [27]. A vitamin B_6_ and sodium bicarbonate solution is convenient to obtain for clinical use. VSBS is a safe and effective contrast agent used for c-TTE that has little side effect. Furthermore, it is applied to the diagnosis of congenital heart disease [14, 15]. Vitamin B_6_ and sodium bicarbonate interact in solution in a traditional acid-base neutralization reaction, which does not result in changes to the structure of vitamin B_6_. The neutralization reaction occurs between the hydrochloride group provided by vitamin B_6_ and bicarbonate when the two chemicals are mixed [15]. When VSBS, which is an affordable agent, was injected, the reaction product is carbon dioxide, which is characteristically safe. VSBS contrast agent does not require a three-way stopcock and exchange to avoid blood splashing that is otherwise caused by an improper exchanging operation. VSBS also avoids the distressing patients, who otherwise are concerned about injection of visible gas into the body. However, the neutralization reaction between vitamin B_6_ and bicarbonate produces more carbon dioxide and a longer microbubbles' peak time, which improves the opportunity to observe and diagnose PFO-RLS [15]. Among a variety of gases that could be used during the right heart contrast echocardiography, carbon dioxide has a superior solubility and diffusion rate. Therefore, carbon dioxide has been found to be the safest for use in the blood [28]. In our study, no patient reported an obvious adverse event during the procedure or during the 12-hour follow-up. Therefore, it is expected that the procedure described herein is the preferred contrast agent for the diagnosis of PFO-RLS.

This preliminary study has several limitations. Poor intolerance among patients for the TEE probe and failure to perform a standard Valsalva maneuver while the TEE was being performed may affect the accuracy of the results to some extent. Another limitation is the small sample size of our study. Further prospective multicenter studies with larger populations are warranted to confirm and extend the conclusions of our study.

Our preliminary study indicated that the application of vitamin B_6_ and sodium bicarbonate solution as a c-TTE contrast agent yielded a higher sensitivity compared to ASB without noticeable adverse events when TEE was used as the reference. Therefore, vitamin B_6_ and sodium bicarbonate solution may be used during contrast-transthoracic echocardiography for the diagnosis of PFO-RLS giving its simplicity, feasibility, and noninvasive nature.

## Figures and Tables

**Table 1:** Baseline characteristics of the study population.

Clinical characteristics	*N* (%)
Sex (male/female)	36/48 (57.0%)
Age (y), mean ± SD	39.2 ± 4.6
Diabetic mellitus	10/84 (11.9%)
Hyperlipidemia	8/84 (9.5%)
Arrhythmia	2/84 (2.3%)
Hypertension	11/84 (13.1%)
Reason for visit	
Ischemic stroke	24/84 (28.5%)
Migraine with aura	20/84 (23.8%)
Migraine without aura	8/84 (9.5%)
TIA	17/84 (20.2%)
Cerebral infarction	15/84 (17.9%)

TIA = transient ischemic attack.

**Table 2:** Diagnostic results of ASB and VSBS compared with those of TEE during the Valsalva maneuver.

VSBS	TEE		Total	ASB	TEE		Total
	Positive	Negative			Positive	Negative	
Positive	49	5	54	Positive	41	4	45
Negative	3	27	30	Negative	11	28	39

Total	52	32	84	Total	52	32	84

ASB = agitated saline plus blood; VSBS = vitamin B_6_ and 6 ml sodium bicarbonate solution; TEE = transesophageal echocardiography.

**Table 3:** Results of the semiquantitative shunt grading using ASB and VSBS during the Valsalva maneuver.

	ASB (*n* = 84)	VSBS (*n* = 84)
Negative	39	30
Positive	45	54
Mild	17	11
Moderate	18	27
Extensive	10	16

ASB = agitated saline plus blood; VSBS = vitamin B_6_ and 6 ml sodium bicarbonate solution.
